# Social Media Metrics and Popular Legitimacy: Content Analysis of Pre– and Post–COVID-19 Public Engagement With the World Health Organization on X

**DOI:** 10.2196/69959

**Published:** 2025-12-08

**Authors:** Thierry Warin, Cristiane Melchior, Nathalie de Marcellis-Warin

**Affiliations:** 1 HEC Montréal Montreal, QC Canada; 2 Lappeenranta-Lahti University of Technology Lappeenranta Finland; 3 Polytechnique Montréal Montreal, QC Canada

**Keywords:** popular legitimacy, World Health Organization, public engagement, social media metrics, COVID-19, computational methods

## Abstract

**Background:**

The World Health Organization (WHO) plays a critical role in global health governance, but its popular legitimacy, a measure of public trust and support, has been contested, particularly during crises such as the COVID-19 pandemic. While legitimacy is widely studied through normative and elite-focused approaches, empirical assessments using public discourse remain limited. Social media platforms like X (formerly Twitter) offer real-time data for evaluating public sentiment toward the WHO.

**Objective:**

This study aims to assess the evolution of the WHO’s popular legitimacy from 2008 to 2021 by analyzing public engagement metrics on X, with a particular focus on changes during the COVID-19 pandemic.

**Methods:**

We analyzed 46,667 tweets from the WHO using computational methods, including the retweet-to-reply ratio, sentiment analysis, and longitudinal trend evaluation. Metrics such as likes, retweets, and replies were examined to quantify public sentiment, with the retweet-to-reply ratio serving as a key indicator of controversy and support levels.

**Results:**

The WHO’s popular legitimacy was stable from 2008 to 2019 but declined significantly during the COVID-19 pandemic, reflecting heightened public scrutiny and criticism. Engagement metrics revealed increased replies relative to retweets during this period, indicating greater controversy in public discourse.

**Conclusions:**

This study demonstrates the feasibility of using social media metrics to measure international organization (IO) legitimacy over time. The findings highlight the impact of global crises on public trust and provide a replicable framework for assessing the legitimacy of other IOs. Social media engagement offers valuable insights for IOs to adapt communication strategies and maintain public trust during crises.

## Introduction

The World Health Organization (WHO) plays a central role in global health governance, coordinating responses to health crises and shaping international health policy. Its legitimacy is crucial for mobilizing resources, fostering international cooperation, and ensuring public compliance, particularly during crises such as the COVID-19 pandemic. A loss of public trust can severely undermine the WHO’s authority and effectiveness. In this context, X (formerly Twitter) offers a valuable platform for analyzing public sentiment, given its role as a real-time medium for discourse and information exchange. Engagement metrics—likes, retweets, and replies—serve as proxies for public reactions, enabling researchers to quantify support, criticism, or disengagement in response to the organization’s communications. These interactions reflect both the reach and the reception of WHO messaging, making X a compelling medium for observing shifts in popular legitimacy over time. Our work builds on prior analyses of media narratives during the COVID-19 pandemic. For example, Chan and Yu [1] examined Chinese state-run media, highlighting themes of cooperation and science to legitimize China’s response; Feifei [2] analyzed metaphors (such as war and family) to show their ideological impact; Yu [3] explored the use of collectivist rhetoric promoting a “Community with a Shared Future for Humankind”; and Bahi [4] analyzed US-China dynamics through Kindleberger’s crisis response framework. Recent research has also shown that public trust in global health institutions like the WHO is shaped by their actions and by how those actions are perceived and debated in digital spaces [5]. Together, these studies underscore how narratives can shape global legitimacy.

Traditional research on organizations like the WHO identifies three main approaches to evaluating legitimacy: (1) survey-based assessments of public opinion, (2) analysis of elite discourse (for example, how political or institutional leaders discuss the organization), and (3) theoretical or offline indicators of legitimacy (such as legal mandates or formal performance metrics) [6,7]. While these methods offer essential insights, they often overlook the real-time, large-scale public discourse occurring on social media. Antonakaki et al [8], for instance, demonstrated the potential of online social networks (OSNs) for analyzing political communication through natural language processing. Yet few studies have leveraged social media to examine the WHO’s legitimacy, and none, to our knowledge, have conducted a longitudinal analysis spanning a decade or more. Our study fills this gap by using X engagement data to track the evolution of public trust in the WHO, particularly during key events such as the COVID-19 pandemic. This approach captures public sentiment at scale, complements traditional legitimacy assessments, and allows for dynamic, event-linked analysis. We focus on the retweet-to-reply ratio as an indicator of public sentiment (a “ratiometric” analysis), drawing on the methodology of Minot et al [9]. Replies often signal dissent or scrutiny, whereas retweets typically indicate endorsement [9]. Thus, a higher ratio of retweets to replies suggests more supportive engagement, while a lower ratio may reflect public skepticism. Notably, this interpretation is echoed in a simple heuristic described by O’Neil [10], who observed that a tweet is often deemed “terrible” if the number of comments it attracts vastly outnumbers its retweets and likes—essentially a case of negative public reception. By tracking the retweet-to-reply ratio over time, we can map fluctuations in the WHO’s perceived legitimacy on the platform.

Legitimacy can be defined normatively as an institution’s moral right to rule, and empirically (popularly) as the public’s belief in that institution’s rightful authority [11,12]. We define popular legitimacy as arising from a belief in the rightful authority of an institution, a belief that can be quantified through observable indicators of public support [13,14]. In the realm of global health governance, legitimacy is increasingly mediated by digital engagement—platforms like X serve as both echo chambers and arenas for testing an institution’s credibility [5]. Echoing this view, we conceptualize popular legitimacy as a form of public trust continuously shaped by ongoing, public-facing narratives, especially during high-stakes periods such as pandemics. A legitimate political authority requires a justifiable basis for power and is seen as authoritative by those it governs, and legitimacy assessments may have policy implications [15].

Our text-as-data approach addresses the first 2 aspects by providing evidence of public justification and acceptance of the WHO’s authority through web-based engagement (the third, policy outcomes, lies beyond our scope). In addition, Gilley [16] pioneered a quantitative approach to legitimacy (categorizing legitimacy into dimensions such as legality, justification, and consent). Our study similarly leverages social media discourse as a real-time lens on legitimacy perceptions, marking a shift from traditional survey measures to engagement metrics derived from behavior on the web. A number of recent studies have examined how international organizations (IOs) cultivate or communicate legitimacy, both offline and online. Hall et al [17] examined how emerging multilateral institutions (eg, the Asian Infrastructure Investment Bank, the Eurasian Economic Union, and various EU bodies) crafted self-legitimating narratives during crises. Similarly, Aagaard [18] and Ecker-Ehrhardt [19] investigated IO communication strategies on social media and urged that more empirical research should consider public-facing legitimacy. Other work has highlighted the disparity between elite and public perceptions of IO legitimacy. Dellmuth et al [20] used cross-national surveys to reveal a persistent gap between how elites and ordinary citizens view the legitimacy of organizations (including the WHO). Elite narratives within IOs evolved over time and were influenced by events such as institutional reforms and crises [21]. While these studies shed light on elite discourse, our focus shifts to the sentiments of the broader public as expressed via social media.

Evidence suggests that the WHO and other IOs have faced growing scrutiny on the web. IOs increasingly lean on democratic and participatory rhetoric when responding to public scrutiny [6]. Yang [22] examined the WHO’s legitimacy crisis during the COVID-19 pandemic, documenting widespread public distrust and criticism. Domestic political divides fuel skepticism toward global institutions like the WHO [23,24]. To quantify public sentiment in such contexts, Minot et al [9] introduced the retweet-to-reply ratio as a measure of audience reaction to political figures—a method we adapt and extend here to evaluate reactions to the WHO’s communications. On a normative theory note, an institution’s legitimacy derives from both public perceptions and its performance [7]. While institutional performance is undoubtedly important, our study centers on the perception aspect (popular legitimacy) by empirically examining public trust over time—addressing a longstanding gap in longitudinal analyses of IO legitimacy on digital platforms. Social media data have become a valuable resource for analyzing public discourse during health crises. Lyu et al [25] and Blane et al [26] analyzed vaccine-related sentiments and community responses on Twitter, while Tahamtan et al [27] studied how well the WHO’s pandemic messaging aligned with public concerns. Pierri et al [28] and Edinger et al [29] tracked the spread of COVID-19 misinformation on X, and Li et al [30] examined public discussions of occupational health on Twitter. However, despite this growing body of work, few studies have systematically used social media engagement metrics to evaluate the legitimacy of an IO.

Our research addresses this gap by offering, to our knowledge, the first longitudinal assessment of public engagement with the WHO’s communications, covering more than a decade of tweets. This long-term perspective provides a unique empirical foundation for observing how the WHO’s popular legitimacy has evolved, particularly through the COVID-19 pandemic and other major global health events. A key methodological consideration in our analysis is how different engagement metrics reflect public sentiment. Each metric captures a distinct form of user interaction, and prior research suggests that retweets and replies are especially informative for gauging sentiment polarity. Following Minot et al [9], who observed that “likes” tend to vary less with content, we emphasize the contrast between retweets and replies as a clearer signal of audience attitude. Indeed, Minot et al [9] found that replies are often associated with dissent or criticism, whereas retweets typically signal support. Consistent with this, Yang [22] observed that replies to the WHO during COVID-19 were filled with distrust and frustration, and Low et al [31] noted that tweets perceived as disagreeable attract disproportionately more replies than likes or retweets. These patterns are corroborated by other findings. Replies to IOs often question the organization’s legitimacy during contentious periods [20]. Replies to public health agencies frequently include critique and misinformation [32] that are characteristic of the broader landscape of COVID-19 fake news on social media [33-35].

In practice, O’Neil [10] offers a rule of thumb: a tweet is “terrible” if it gathers far more comments (replies) than retweets or likes. All of these insights reinforce the use of the retweet-to-reply ratio as a barometer of public sentiment—where a high ratio indicates broad endorsement of a WHO message, and a low ratio signals widespread skepticism. In summary, conceptually we show that an IO’s legitimacy can be traced through its digital engagement patterns, and methodologically we provide a framework to monitor public trust via social media data. The 13-year span of our dataset allows us to observe trends and correlate shifts in legitimacy with major events. Additionally, we tested whether the positivity of the WHO’s own messaging influenced public engagement; a sentiment analysis found no significant correlation (Multimedia Appendix 1), suggesting that declines in public support were not driven by changes in the tone of the WHO’s tweets. Accordingly, our study is guided by the following research questions (RQs):

- RQ1: How did the popular legitimacy of the WHO evolve between 2008 and 2021, as reflected by public engagement on X?
- RQ2: What impact did the COVID-19 pandemic have on the WHO’s popular legitimacy, as observed through changes in public engagement metrics on X?
- RQ3: Can public engagement metrics—particularly the retweet-to-reply ratio, supplemented by sentiment (positive vs negative reactions)—serve as reliable indicators of the WHO’s popular legitimacy?
- RQ4: How does the WHO’s pattern of engagement on X reflect broader trends in its legitimacy and the public’s response over time?

To answer these questions, we adopt a longitudinal computational analysis of the WHO’s presence on X. In particular, we introduce a novel approach for assessing the WHO’s popular legitimacy by tracking its public engagement metrics over a 13-year period. Drawing on social media analytics techniques, we implement an empirical protocol that monitors legitimacy signals (endorsement vs dissent) through engagement indicators. Our dataset comprises 46,667 tweets from the WHO’s official account, covering the period from April 23, 2008 (the date of the WHO’s first tweet), to November 8, 2021. This dataset encompasses the entirety of the organization’s activity on the platform during that time frame, providing a comprehensive basis for our longitudinal analysis.

## Methods

### Theoretical Background

Because the responses to each original activity include timestamped counts for a variety of metrics, such as replies, likes, and retweets, it is possible to create a historical timeline of events using this information. We use this timestamp to create daily aggregate measures when necessary. As in Minot et al [9], when the term “activity” is used in this study, it refers to any user action that has been documented in the historical sample, including original tweets, retweets with comments, and answers to those tweets.

Thanks to this historical timeline, we can look at counts but also changes in the various count measures. We examine the second derivative of the various activities (retweets, replies, and likes), as doing so will help us capture the acceleration or deceleration of an activity. Specifically, we focus on the second derivative of retweets, as the literature favors this indicator to measure the degree of interest in a conversation [9].

To empirically examine the characteristic time scale of response activities, we present the time index *t* corresponding to the local maxima of the instantaneous retweet count for each tweet, defined as the local maxima of the first derivative of cumulative retweets *N*_retweets_, or 
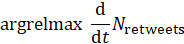
. To conduct an empirical investigation of the distinctive time scale of response activities, we look for occasions where the second derivative of retweet counts decreases below zero,



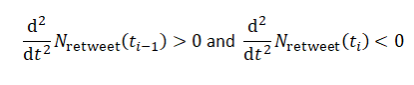



because new activities are created at a decelerating rate, these points indicate that response activity is beginning to diminish or “roll over.”

To demonstrate the ternary ratio values, a ternary plot (2D simplex) is used in which the values of activities at each time step add up to one at the end of each time step.



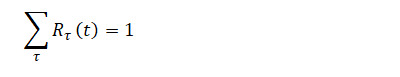



We will standardize our approach by looking at each activity in relation to the overall sample of activities. Temporal activity values are derived by dividing each activity count by the sum of all the activities at a given time step in a certain time step. For activity type *τ* at time step *t*, the ternary ratio value may be calculated as:



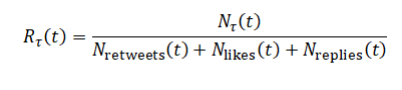



where *N_τ_*(*t*) is the number of times the activity occurred at the time step *t*. A 3D vector reflecting the normalized activity values associated with a tweet is created for each observation in the manner described above [9].

Several potential shortcomings in our methodological approach should be noted. Since our technique is entirely observational, we cannot rule out the possibility that it contains certain flaws: for example, conversations outside our sample may have an impact on the dialogues and themes posted by the WHO. Our sample includes the whole population of tweets from the WHO, but does not include the entire population of tweets concerning a public health debate. Next, we are unable to control for homophily (ie, the propensity to connect with individuals who share similar traits [36]) in other information sources. For example, the sample may be skewed because of a low rate of survival.

**Figure 1:**
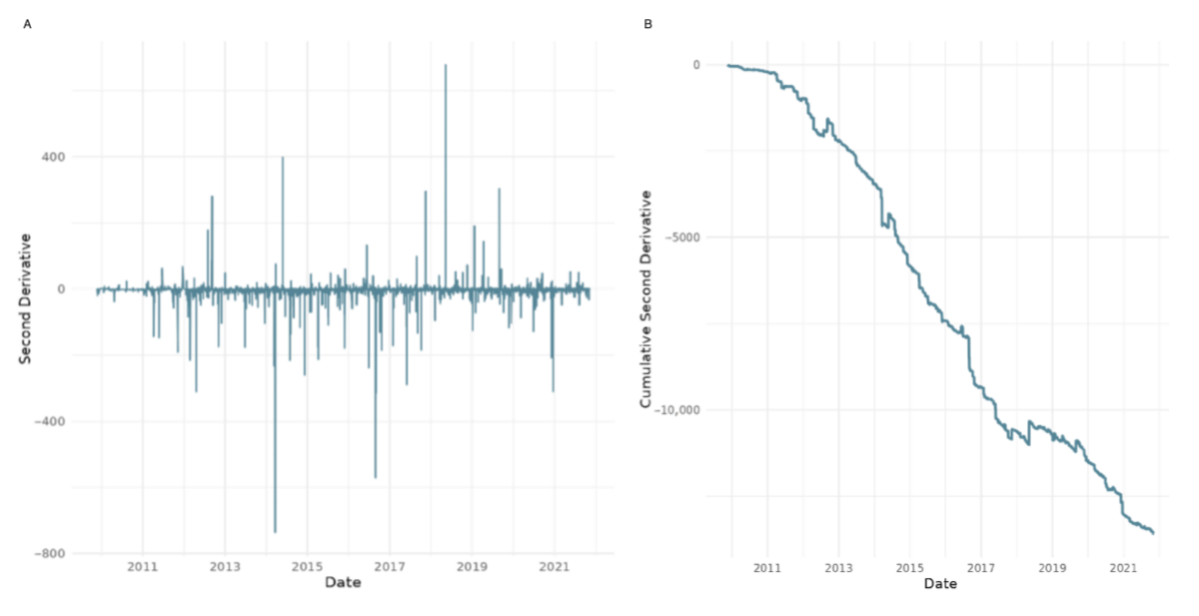
Characteristic time scale of audience engagement with World Health Organization tweets over the entire period of the study (2008-2021).

**Figure 2:**
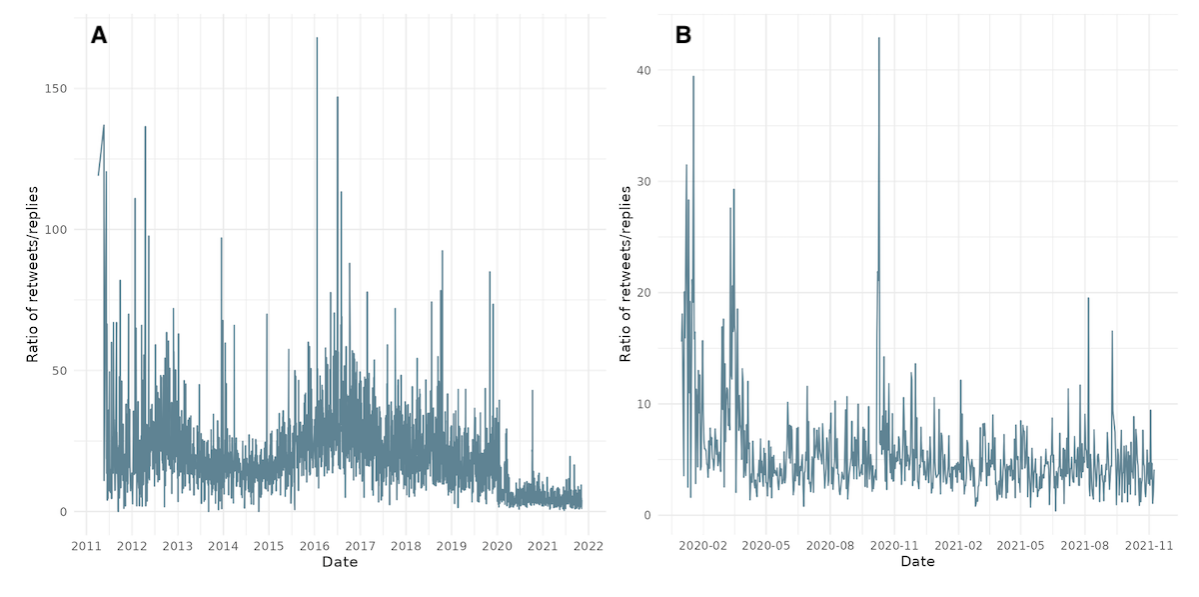
Daily retweet-to-reply ratio for the World Health Organization’s tweets: (A) 2011-2021 and (B) COVID-19 period 2020-2021.

Another potential shortcoming is the concentration we place on the 2020-2021 period. While COVID-19 was by far the most impactful pandemic in the time frame we consider, other pandemics and events impacting the health of populations have had large, albeit temporary, impacts on the popular legitimacy of the WHO. For example, the Ebola outbreak in mid-2014 and the Rohingya refugee crisis of mid-2018 both clearly showed positive spikes (Figure 1) that incited much conversation. However, as evidenced by the same figure, these events do not appear to have caused any long-term changes in the popular legitimacy of the WHO. On the other hand, notable, permanent differences in certain metrics (eg, the retweet-to-reply ratio in Figure 2) began in 2020. For this reason, we chose to focus our analysis on the comparison of the years prior to COVID-19 and those in which the pandemic was present.

We acknowledge that our analysis cannot account for passive indifference, those who ignore or disengage from WHO communications. While this remains a limitation, declining engagement (eg, fewer likes and retweets without a corresponding rise in replies) may suggest growing apathy. Such trends, which could stem from reduced WHO activity or broader shifts in platform use, fall outside the scope of this study but warrant future investigation.

According to previous research [9,20,22,31,32], we also implicitly assume that replies are negative. While this is not true for all replies, it is a relatively safe assumption, especially for the X accounts of IOs. On these accounts, personal interactions, which are more likely to be positive, are rare.

Finally, we are unable to directly capture those who ignore tweets or are indifferent to the communications of the WHO. While this remains a shortcoming, we may still gain an idea of the extent to which this happens indirectly through decreases in any (or all) of the measures. For example, a decrease in retweets and likes not being met with a proportional increase in replies may be a sign of increased indifference among users. More generally, an overall decrease in interactions could be the result of several phenomena, such as a general decrease in user activity on X or a lower frequency of tweeting from the WHO account. These possibilities are beyond the scope of our paper and should be addressed in future work.

### Data Collection

X’s response volume is perhaps the most useful measure for determining how the platform’s user base reacts to new content. In addition, these values are immediately obvious to users, which may affect their behavior if they wish to impact the collective reaction to a communication. Understanding how users react to varying levels of activity volumes and temporal dynamics necessitates first developing some discrete scales on which to measure their behavior [9].

Several other indicators are also worth considering. The number of persons who have reacted to a given tweet is indicated by symbols at the bottom of each tweet that also enable users to communicate with one another through an interface. These numbers serve as a measure of a tweet’s popularity or controversy, depending on the context. With the original tweet’s cultural context in mind, the ratio of these factors may be used to evaluate tweets using “ratiometrics,” which refers to the analysis of ratios between X engagement metrics (replies, retweets, and likes) as a means of characterizing audience reactions to tweets [31]. It may be possible to reduce the response activities of the user base to aggregated measurements of their reactions as a consequence of this reduction, which also enables the comparison of X response habits between accounts and over time. Next, we can begin to examine tweets based on their content by referring to the ratio data as a guideline [9].

Calculating public reaction activity counts provides a useful starting point, but does not fully capture the potential of ratiometrics, as demonstrated in this study. The “ratiometer” is a suite of analytical tools designed to quantify and interpret response activity ratios—such as replies, retweets, and likes—offering insights into public engagement dynamics and enabling comparative evaluations across users and periods [9]. For accurate analysis, it is essential to account for a user’s typical activity ratios and the age of the tweet. It is critical to understand the standard response patterns for an account and the expected reaction volume over time since a tweet’s publication to refine the interpretation of social media activity [9]. The tweets and associated metadata (date, username, retweets, and hashtags) of the WHO have been gathered for this study since the organization first began tweeting.

We do not execute any content tampering or re-engineering as part of our protocol, which consists merely of monitoring the X stream (with no information filtering, prioritizing, ranking, or any other process) [37]. The WHO’s posts serve as our null model against which we can measure the number of likes, responses, and retweets received. This approach allows us to test the legitimacy of the theory by studying the responses of various individuals to different narratives on the internet.

The data were acquired using an R-based application that queried the streaming application programming interface of the X service. It comprises 46,667 tweets sent between April 23, 2008, and November 8, 2021, according to the dataset. The commencement date corresponds to the first tweet sent out by the WHO.

### Data Analysis

First, we gained a comprehensive understanding of the count of retweets and replies by examining their cumulative function. Second, we calculated the retweet-to-reply ratio to gauge the level and type of audience engagement. Third, we validated the second step’s conclusions by analyzing the retweet-to-reply ratio’s cumulative function.

### Ethical Considerations

This study was conducted in accordance with all relevant ethical guidelines. The research is based exclusively on publicly available data retrieved from the official, verified X account of the WHO. The dataset consists of the WHO’s original tweets and the corresponding aggregated engagement metrics, specifically, the counts of likes, retweets, and replies.

All data used in our analysis were fully deidentified. We did not collect, analyze, or store any personal information from individual X users, such as usernames, profile details, or the content of their replies. The focus of the analysis was on the collective, aggregated response patterns to the WHO’s public communications.

Given that the study relies entirely on publicly accessible and nonidentifiable data, it does not involve human participants in a manner that would necessitate formal review by an institutional review board, and was therefore exempt from such approval. Data collection and handling were performed in strict compliance with X’s terms of service and data availability policies. Accordingly, this research poses no direct risk to individuals.

## Results

### Overview

We applied these 3 steps to the entire dataset and then focused specifically on the COVID-19 years, 2020 and 2021, to explore the impact of the pandemic on the WHO’s legitimacy as reflected in its X engagement.

### Step 1: The Cumulative Sum of Retweet and Reply Activities

**Figure 3:**
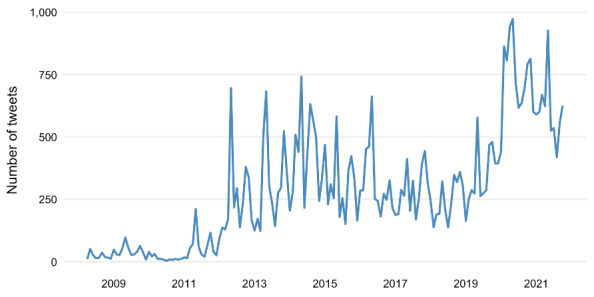
Number of tweets published monthly by the World Health Organization on X from 2008 to 2021.

Figure 3 presents the number of tweets published by the WHO on X from 2008 to 2021. A marked inflection point is evident in early 2020, corresponding with the onset of the COVID-19 pandemic, when tweet frequency increased sharply.

**Figure 4:**
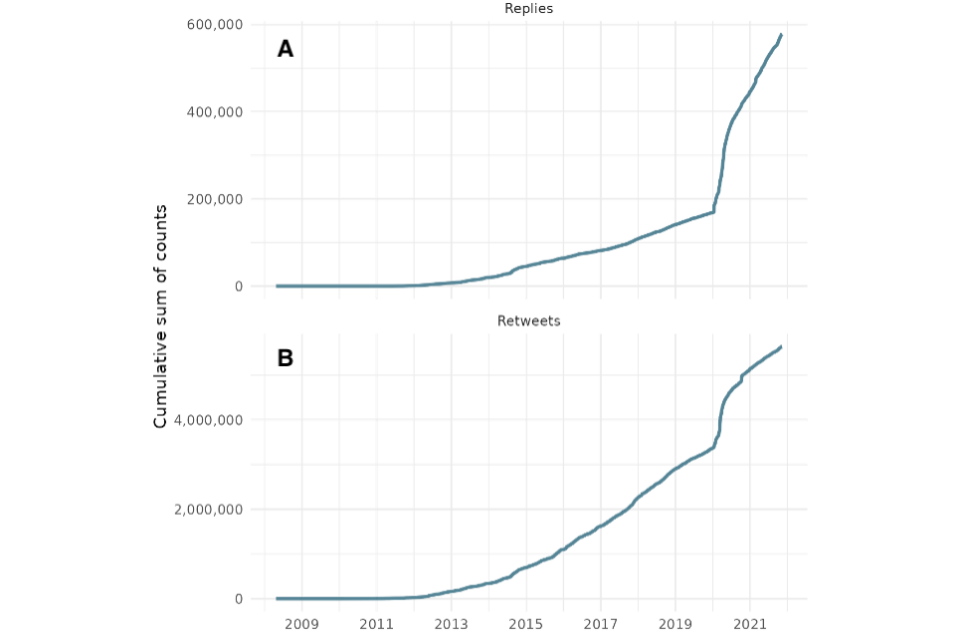
Cumulative engagement with (A) retweets and (B) replies published by the World Health Organization on X from 2008 to 2021.

Figure 4 illustrates the cumulative number of replies (Figure 4A) and retweets (Figure 4B) to WHO posts on X, aggregated daily from 2008 to 2021. The y-axis represents the cumulative count of interactions, while the x-axis denotes time in years. Similar to the trend in Figure 3, both replies and retweets show a pronounced spike during the early months of the COVID-19 pandemic. Notably, the cumulative curve for replies rises more steeply than that for retweets during this period, indicating intensified user engagement and possibly increased public concern or debate. These patterns suggest a relevant shift in public interaction with WHO communications during the global health crisis, underscoring how digital discourse intensifies in response to emergencies.

**Figure 5:**
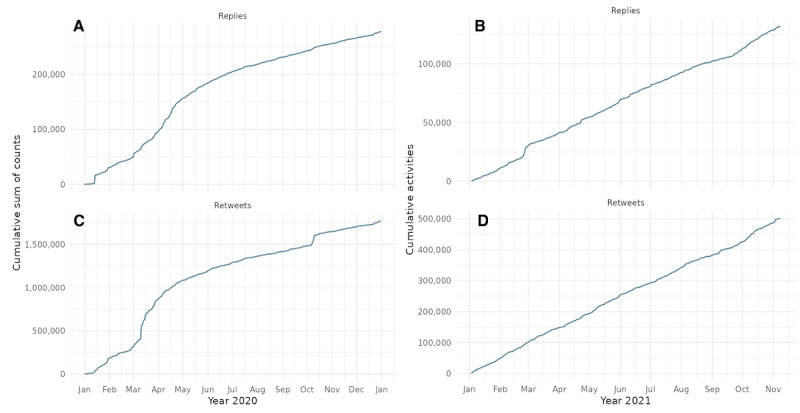
Cumulative count of public engagement: (A,B) replies and (C,D) retweets, with World Health Organization tweets over 2020 and 2021, respectively. Engagement is used as a proxy for public attention and sentiment, contributing to real-time legitimacy assessments.

Figure 5 offers a more granular view of user engagement during 2020 (left) and 2021 (right), illustrating the cumulative sum of replies and retweets to all WHO tweets, separately by engagement type. The steep slope observed in early 2020 reflects a surge in public attention following the emergence of COVID-19. Both replies and retweets rise sharply in March and April 2020, aligning with the WHO’s pandemic declaration on March 11, 2020, and escalating global uncertainty. This trend indicates increased visibility and scrutiny of WHO messaging, with the sharper rise in replies potentially signaling critical engagement or questioning from the public. In contrast, engagement in 2021 follows a more gradual, linear trajectory, retweets largely plateau, and replies show only modest increases, with a minor spike observed in March 2021.

We recognize that cumulative activity measures (such as cumulative likes, retweets, and replies) are influenced by changes in public sentiment and by exogenous factors such as the expanding user base and increased penetration of X over time. This temporal drift, particularly during the pandemic, may amplify activity trends in ways that are not solely attributable to shifts in perceived legitimacy. Thus, we interpret these cumulative indicators primarily as background context and rely more heavily on normalized metrics, such as the retweet-to-reply ratio and ternary engagement composition, to assess relative changes in public engagement and sentiment.

In addition, the cumulative plots, represented in Figures 4 and 5, are included to contextualize the overall volume and timing of user interactions with WHO tweets. While these figures highlight relevant surges in activity, particularly during key pandemic phases, they are not used as direct evidence of shifts in popular legitimacy due to known limitations in interpreting volume-based metrics over time. For legitimacy assessment, we rely on ratio-based indicators that normalize for scale and better account for platform growth and shifting user demographics.

### Step 2: Retweet-to-Reply Ratio

As previously mentioned, the retweet-to-reply ratio is a well-established metric in the literature, as its evolution serves as a reliable proxy for changes in the positive perception of an organization’s post. The underlying rationale of this metric is that retweets generally indicate agreement or support for the content, while replies often suggest a desire to engage in conversation, potentially expressing disagreement or criticism. Consequently, the retweet-to-reply ratio can be interpreted as follows: the higher the ratio, the less controversial the topic and the more positively it is perceived by the audience.

Figure 2 illustrates the evolution of the retweet-to-reply ratio, a key metric used to assess shifts in the WHO’s popular legitimacy on X. The ratio is calculated by aggregating the number of retweets and replies received by the WHO’s tweets each day and dividing the total daily retweets by the total daily replies. A higher ratio suggests tweets were more widely endorsed (via retweets) relative to critique or discussion (via replies), implying stronger perceived legitimacy or reduced controversy. Conversely, a lower ratio indicates increased scrutiny, criticism, or contentious engagement. The left panel presents daily fluctuations over the full observation window (2008-2021), while the right panel isolates the pandemic period (2020-2021), showing a sustained drop in the ratio beginning in early 2020. This decline suggests a notable shift toward more critical engagement with the WHO during the COVID-19 pandemic.

In Figure 2, we notice a decrease in the retweet-to-reply ratio, which corroborates the initial descriptive statistics from earlier figures that indicate a decrease in popular legitimacy coinciding with the beginning of the COVID-19 pandemic. To gain a more accurate understanding of this trend, we analyzed the cumulative ratios. This approach allowed us to identify whether changes in the ratio are indicative of long-term trends or short-term fluctuations and better understand the implications for the organization’s popular legitimacy.

### Step 3: Cumulative Function of the Retweet-to-Reply Ratio

**Figure 6:**
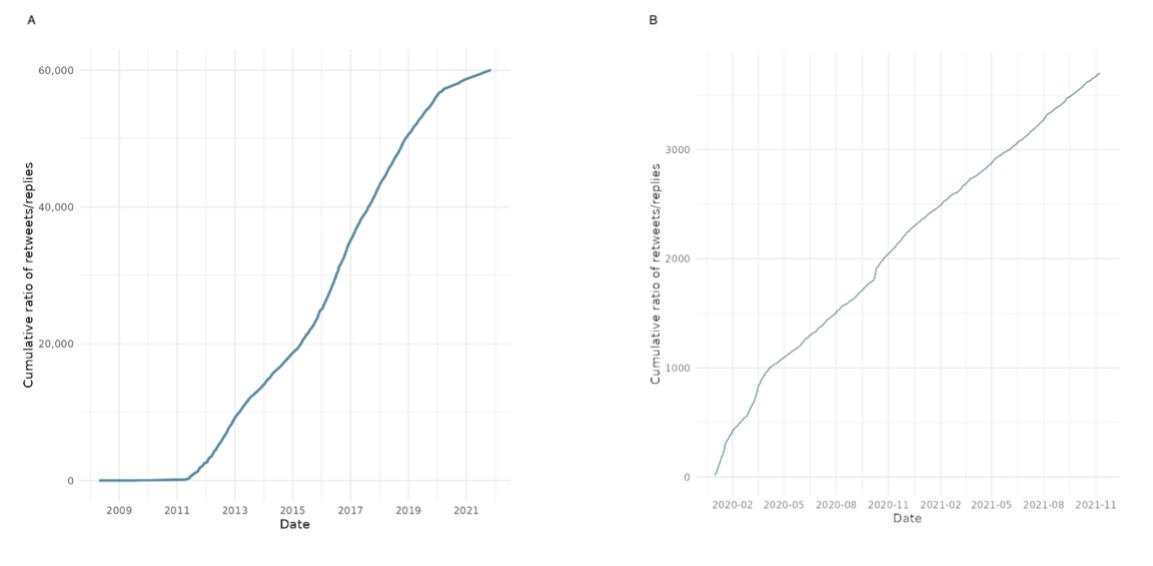
Cumulative retweet-to-reply ratio for the World Health Organization’s tweets: (A) 2008-2021 and (B) for the COVID-19 period 2020-2021.

Figure 6 presents the cumulative evolution of the retweet-to-reply ratio as a proxy for popular legitimacy in response to WHO communications on X. The metric is computed by aggregating daily retweet and reply counts across all WHO tweets and calculating the running total of retweets divided by the running total of replies over time. Days with undefined or infinite values (eg, zero replies) were excluded to ensure analytical validity. The left graph illustrates the engagement across the 13-year period studied, showing a largely stable ratio until early 2020. Starting in the COVID-19 period (2020-2021), there is a marked and sustained decline in the cumulative ratio, indicating an increase in critical or controversial responses relative to supportive engagement. The right graph shows this period in more detail.

The above figure illustrates the impact of COVID-19 on public engagement metrics with the WHO. The flattening of the cumulative curve in 2020 and 2021 indicates a reduction in the growth rate of the retweet-to-reply ratio compared with previous years. This demonstrates a higher engagement of the public receiving the information disseminated by the WHO, although it is likely seen in a more negative light.

In addition to engagement-based metrics, we conducted a sentiment analysis of WHO tweets using the AFINN lexicon from the tidytext R package. The sentiment analysis allowed us to track changes in emotional tone across the dataset, particularly in the context of the COVID-19 pandemic. Both daily sum and mean sentiment scores were calculated to observe net positive or negative shifts over time (Multimedia Appendix 1 contains more details).

Our dataset enables investigation at several different levels. For example, we can see the rate (first and second derivatives) at which the WHO tweets, which is mainly linked to recent global health crises. Below, we discuss the COVID-19 period in further detail. It is possible to determine which subjects have been retweeted and liked by the public by analyzing the dynamics of the dialogues and issues promoted by the WHO on X.

The initial step involves completing an exploratory data analysis that is primarily focused on volumes: the total number of tweets made by the WHO, followed by the total number of likes, replies, and retweets. Despite covering only 11 of 12 months in 2021, we notice a substantial spike in the number of tweets made by the WHO in 2020; in 2021, this number decreases to levels similar to those of prior years.

The analysis becomes more nuanced when focusing on engagement metrics such as likes, retweets, and replies. We observe that a significant number of tweets posted by the WHO engaged the audience, as indicated by the engagement metrics. Specifically, 44,682 tweets were retweeted at least once, reaching a maximum of 52,439 retweets for a single tweet. This high level of retweeting suggests a broad dissemination of the WHO’s tweets across the platform, indicating the content’s relevance and the audience’s willingness to share it within their networks.

Furthermore, 41,881 tweets elicited at least one reply, with the number of replies for a single tweet peaking at 15,180. This considerable amount of interaction signifies active engagement, showcasing that the WHO’s content not only reaches a wide audience but also prompts discussions, questions, or debates, highlighting the conversational aspect of its social media presence.

Likewise, the like metric reveals that 44,100 tweets received at least one like, with the highest number of likes for a single tweet being 53,831. Likes, as a form of engagement, indicate approval of or agreement with the content shared. The significant volume of likes underscores the positive reception of the WHO’s tweets by the audience (Figure 4).

Table 1 shows the number of tweets, retweets, responses, and likes received per year. It provides a chronological overview of the WHO’s engagement metrics on X between 2008 and 2021. The table categorizes data into tweets, likes, retweets, and replies received each year, offering insights into the evolution of public engagement with the WHO’s communications on this social media platform.

**Table 1:** Number of tweets, retweets, replies, and likes per year.

Year	Tweets	Likes	Retweets	Replies
2008	199	16	4	3
2009	513	373	135	1
2010	170	348	2939	0
2011	723	2309	20,638	765
2012	2991	16,087	139,731	6467
2013	3737	30,509	177,663	12,438
2014	5090	122,541	355,772	25,609
2015	3718	204,364	404,247	18,694
2016	3882	345,566	520,396	17,670
2017	3445	637,237	639,682	26,846
2018	2986	831,974	640,625	32,478
2019	4113	782,309	466,499	27,572
2020	8894	3,725,855	1,769,577	278,107
2021	6206	1,310,300	500,999	131,937

In 2008, during a nascent phase of social media engagement, the WHO’s presence on X was modest, and there were minimal likes, retweets, and replies. This trend of relatively low engagement continued until a significant uptick occurred in 2011, when all metrics saw substantial increases, particularly retweets, which surged from 135 in 2009 to 20,638 in 2011. This period marks the beginning of an exponential growth in engagement, reflecting either an increase in the WHO’s activity on X, a growing audience, or heightened public interest in health topics.

The years 2014 and 2015 represent another leap in engagement, with likes and retweets increasing dramatically, suggesting an enhanced recognition of the WHO’s content and its importance among the X user base. By 2017, the numbers for likes and retweets reached new heights, indicating a robust engagement with the WHO’s tweets, which could be attributed to the organization’s authoritative role in information dissemination, especially in times of health crises or significant public health interest stories.

The year 2020 stands out due to the COVID-19 pandemic, with the number of tweets doubling from the previous year and the engagement metrics for likes, retweets, and replies skyrocketing. This surge is likely driven by the global population’s demand for reliable information about the pandemic, demonstrating the crucial role of the WHO in providing timely and accurate public health information during a global crisis. The numbers for 2020 reveal the scale of interaction and widespread reliance on the WHO for guidance during the pandemic, with likes surpassing 3 million, retweets nearing 1.8 million, and replies exceeding 278,000.

In 2021, while the numbers decreased somewhat from the peak year of 2020, they remain significantly higher than prepandemic levels, indicating a sustained engagement with the WHO’s content on X. This continued high level of interaction may reflect the ongoing concerns and discussions about the COVID-19 pandemic and its aftermath, as well as the public’s continued reliance on the WHO for public health information.

Regarding the language distribution of the tweets published by the WHO over 13 years, our dataset comprises 46,667 tweets posted from the WHO’s verified X account, covering at least 23 different languages. English overwhelmingly dominates the corpus, accounting for over 98% of all tweets (n=45,785), reflecting the WHO’s default global communication strategy. In addition, less than 0.5% of the tweets were published in each of the following languages: Spanish, French, Arabic, Indonesian, and Russian.

**Figure 7:**
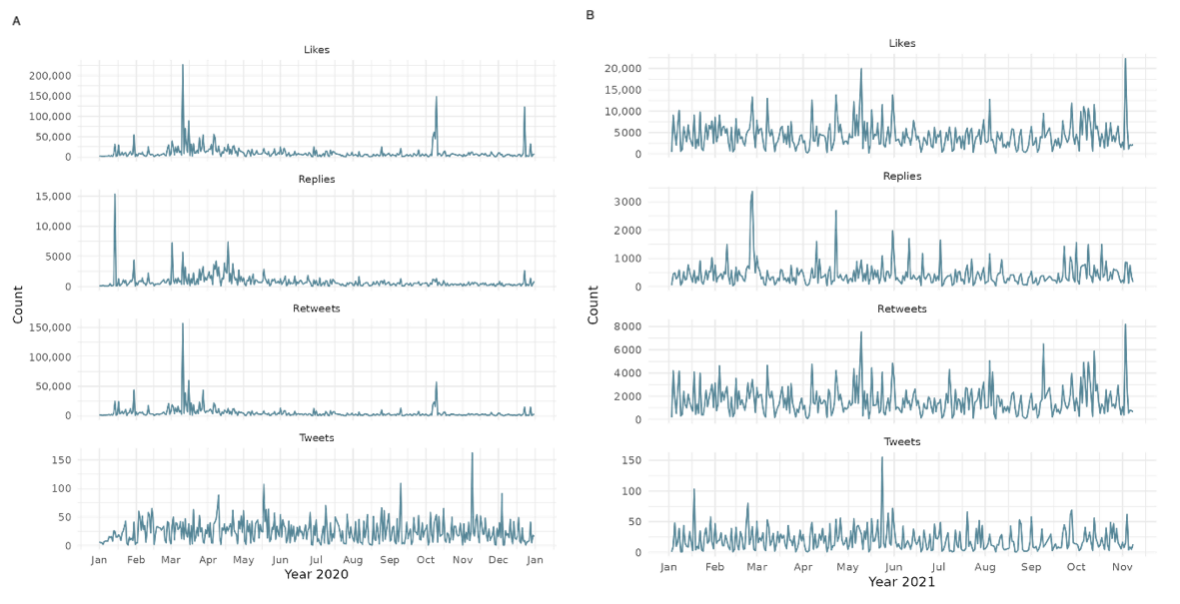
Daily X activity during the COVID-19 pandemic in (A) 2020 and (B) 2021.

Figure 7 plots 4 distinct types of engagement*:* likes, replies, retweets, and tweets per day in the years 2020 (left graph) and 2021 (right graph). Each row represents a different metric of user interaction on the platform: (1) likes (top row), (2) replies (second row), (3) retweets (third row), and (4) original tweets (bottom row). The y-axis denotes the daily count for each interaction type, while the x-axis indicates the time progression from January to December. The data reveal notable surges in engagement throughout 2020, particularly in January, March, April, and November, coinciding with major global and pandemic-related events. A more distributed and frequent pattern of spikes is observed in 2021, indicating a shift in engagement dynamics as the pandemic progressed. Thus, Figure 7 focuses on the final 2 years of our dataset, corresponding to the core period of the COVID-19 crisis, and highlights the temporal variation in public discourse and interaction on X.

### The WHO’s Popular Legitimacy Is Based on X Communications

We provide a visualization of the indicators to measure legitimacy. To this end, we took 3 additional steps.

In the subsequent analysis, we present various metrics derived from the equations discussed above to further investigate the dynamics of audience engagement with the WHO’s communications.

Figure 1 illustrates a temporal analysis of audience engagement dynamics with the WHO’s tweets on X, focusing on the characteristic time scale of public response. The metric is derived from the second derivative of the retweet count time series, an analytical technique adapted from computational social science, which captures the acceleration or deceleration of engagement over time. The left panel reflects fluctuations in retweet activity across the dataset, identifying peaks that correspond to surges in user attention. The right panel shows the cumulative second derivative of retweets, enabling the detection of inflection points when user engagement begins to slow. These turning points serve as indicators of when conversations begin to lose impulse.

This approach allows us to assess not just the volume but the time and persistence of public attention in response to WHO communications. Notably, during the COVID-19 pandemic, the frequency of acceleration-deceleration cycles increased, suggesting heightened reactivity and volatility in user responses, key signals of a contested legitimacy environment. The analysis offers a novel perspective on how audiences temporally engage with WHO during periods of global crisis.

**Figure 8:**
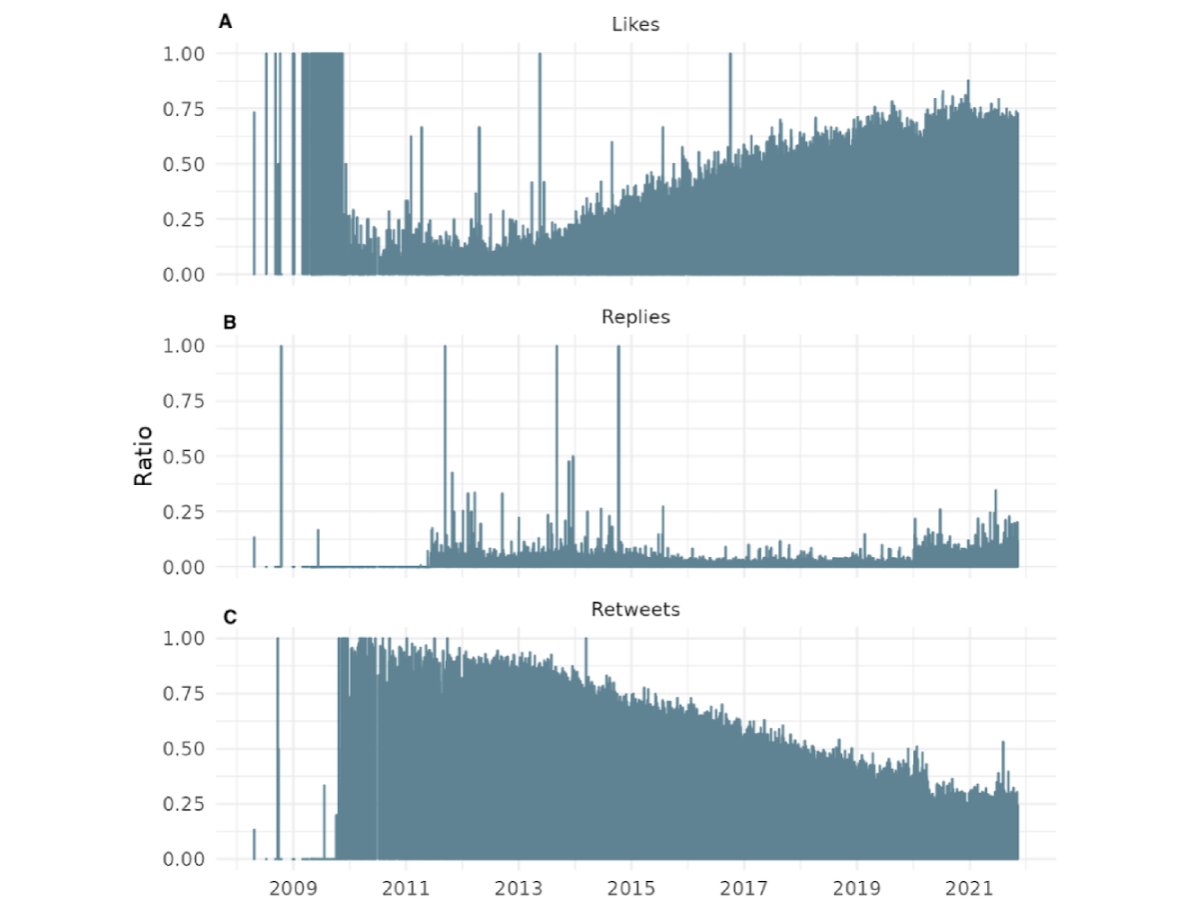
Ternary composition of engagement types (likes, replies, and retweets) in World Health Organization tweets over the full study period (2008-2021).

Figure 8 illustrates the ternary ratio of user engagement metrics (likes, retweets, and replies) aggregated over time to assess how the composition of public responses to the WHO’s tweets evolved. Each point in the ternary plot represents the normalized proportions of the 3 engagement types at a given time step, with all values summing to 1. Between 2008 and 2019, the number of replies remained low and relatively stable, especially during the final 3 years. However, this measure sharply increased around the first quarter of 2020 when the COVID-19 pandemic became worldwide. For the rest of 2020 and 2021, the numbers of likes, retweets, and replies remained relatively stable. One empirical challenge with this evolution is how to interpret the value of a like versus that of a retweet. While we prefer the retweet-to-reply ratio, which decreased through most of the sample period, it must be noted that the decrease in retweets was largely mirrored by an increase in likes. It is only at the start of the pandemic that the retweet-to-reply ratio attains its lowest value—and for the rest of the 2020-2021 period, the decline in retweets is clearly compensated for not by an increase in likes but by an increase in replies.

**Figure 9:**
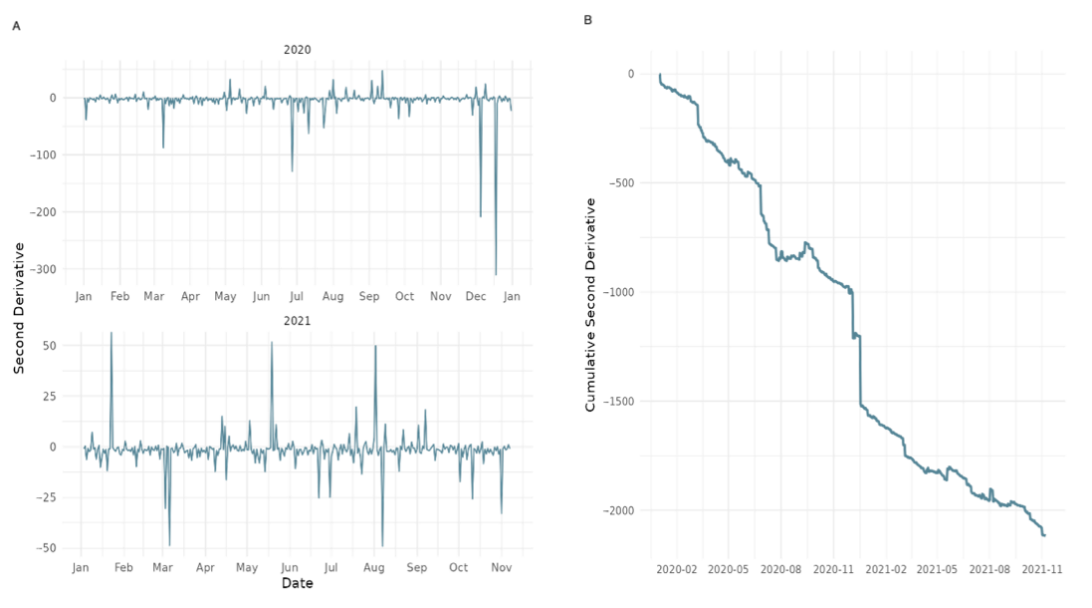
Characteristic time scale of user engagement with World Health Organization tweets during the COVID-19 pandemic (2020-2021).

Figure 9 analyzes the temporal dynamics of audience engagement with the WHO’s tweets during the COVID-19 crisis by focusing on the characteristic time scale. The analysis is based on the second derivative of the retweet count distribution, capturing the acceleration and deceleration of engagement over time. The graph identifies inflection points, moments when user engagement intensity begins to decline, highlighting periods where public attention shifted or waned. Compared with the prepandemic period (Figure 1), the frequency and intensity of these shifts increased substantially in 2020-2021, indicating greater volatility and reactivity in how users responded to WHO communications. This suggests that the public’s attention during the pandemic was elevated and more fragmented and reactive, consistent with the broader observation of contested legitimacy.

**Figure 10:**
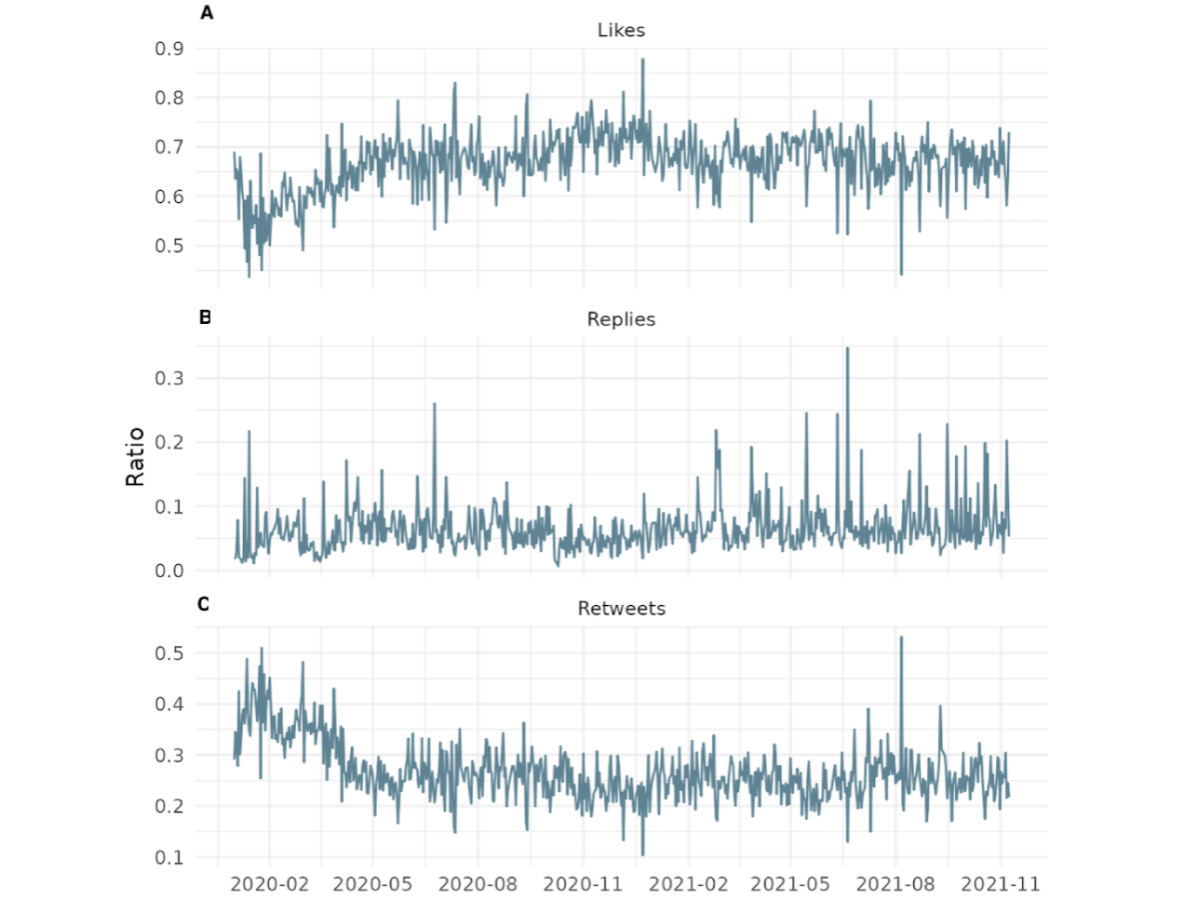
Ternary ratio of likes, replies, and retweets in World Health Organization tweets during the COVID-19 pandemic (2020-2021).

Figure 10 illustrates the ternary ratio of public engagement types (likes, retweets, and replies) with the WHO’s tweets during the COVID-19 period. Each data point represents the normalized distribution of these 3 interaction types at a specific time, summing to a total of 1. This ternary representation provides a comparative view of how different forms of public engagement fluctuated during a period of heightened scrutiny. The data show a notable shift toward replies, particularly in early 2020, coinciding with the WHO’s declaration of COVID-19 as a global pandemic.

### Sentiment Trends Pre- and Post the COVID-19 Pandemic

To complement engagement metrics, we conducted a sentiment analysis of the WHO’s tweets using the AFINN lexicon, which assigns scores to words ranging from –5 (strongly negative) to +5 (strongly positive). We calculated both the daily sum and mean sentiment scores across the entire dataset and compared trends between pre–COVID-19 and COVID-19 era communications (2008-2019 vs 2020-2021). The analysis revealed that sentiment became more positive on average during the pandemic period, particularly after mid-2020. However, this increase in positive tone was not associated with greater engagement acceleration. Indeed, despite the WHO’s more positive messaging, our acceleration analysis (via second derivative of retweets) showed limited impact. These findings suggest that increased positivity in institutional messaging does not necessarily translate into higher engagement or perceived legitimacy. Please note that the full details, figures, and methodological notes are provided in Multimedia Appendix 1.

## Discussion

### Principal Findings

This study introduced an empirical approach to assessing the popular legitimacy of the WHO by analyzing public engagement on X (formerly Twitter) over 13 years. Using a dataset of 46,667 tweets published between 2008 and 2021, we applied computational social science methods to examine how engagement metrics (likes, retweets, and replies) evolved over time as indicators of public sentiment. Our work indicates that the public legitimacy of the WHO remained stable between 2008 and 2019, despite some contestation during the swine flu [38]. According to the previous literature, the stability of an IO’s public legitimacy can be explained by political stability and institutional maturity [39]. Popular legitimacy constitutes a fundamental asset for IOs and one that is essential in acquiring access to requisite resources. In turn, legitimacy is achieved by steadily meeting public expectations [40].

Our findings reveal major trends in public engagement and the perceived legitimacy of the WHO, aligning with prior research [1,3]. While previous studies have primarily examined China’s promotion of official ideologies, national image, and international relations during the COVID-19 pandemic through discourse analysis in newspapers [1,3], our study diverges by focusing on the WHO’s communication strategies on platform X both prior to and during the pandemic.

Moreover, we demonstrated a decline in the WHO’s popular legitimacy during the COVID-19 period. The past literature points to political reasons as a possible explanation for this decline [22]. During the COVID-19 pandemic, the Trump administration accused the WHO of being compliant and of delaying and inadequately handling problems with Beijing [22]. The United States subsequently removed its financial support and canceled its membership in the WHO. The critiques of the Trump administration, along with the simultaneous politicization of the agency and emphasis on its structural deficiencies, resulted in “a concerning loss of confidence in the WHO at a time when the world needs it the most” [41]. During the COVID-19 pandemic, the understanding that the WHO offered privileged treatment to China and pharmaceutical companies negatively influenced the IO’s popular legitimacy [42]. Thus, prior scholarly works have demonstrated the link between politicization and the reduction of IOs’ legitimacy [6].

While Minot et al [9] show that US presidential accounts experienced short-lived “ratio storms,” the WHO’s downturn was both sharper and more persistent. We attribute this difference to two main factors: (1) the trans-sovereign nature of the WHO, which limits its ability to counteract national framing contests [23], and (2) the diffusion of pandemic blame assignments documented by Schmidtke [21]. Our finding that replies, rather than retweets, drive the shift is consistent with the work of Jamison et al [32] on public-health misinformation, reinforcing the idea that critical engagement is clustered in the reply layer.

In addition, our results showing a decline in public endorsement of the WHO during COVID-19 through engagement ratios and sentiment shifts are consistent with the content-driven analysis of Gupta et al [5], which documented growing digital skepticism toward the WHO during the same period. Reputational legitimacy can erode even in the absence of direct institutional failure, especially when digital narratives amplify perceived inaction, politicization, or alignment with contested actors, factors that our engagement and sentiment metrics also capture [5].

The main contribution of this paper lies in its innovative approach to assessing the popular legitimacy of an IO, specifically, the WHO, through X engagement metrics over an extended period (2008-2021). This contribution addresses a key research gap in the literature. While legitimacy is widely discussed in relation to IOs, most existing studies focus on normative legitimacy (the standards IOs ought to meet) or elite views of IO legitimacy. There has been little empirical research on popular legitimacy (public trust and perception) as reflected in real-time public discourse, especially on social media platforms. In addition, very few studies have used a long-term, quantitative approach to examining how the public legitimacy of IOs fluctuates over time and under stress from global crises such as COVID-19.

Thus, we highlight four main contributions. (1) This paper is one of the first to use social media metrics (likes, retweets, and replies) on X (formerly Twitter) to quantify popular legitimacy for an IO over 13 years. By examining patterns of public engagement with the WHO’s communications, the paper introduces a novel, empirical approach to gauging public sentiment toward the WHO, which is an indicator of its perceived legitimacy. (2) Our research uniquely captures changes in popular legitimacy before and during the COVID-19 pandemic. By doing so, it highlights how a major global health crisis impacted public trust in the WHO and gives insights into how IOs are perceived during periods of intense public scrutiny and uncertainty. (3) Our work validates the use of engagement metrics—particularly the retweet-to-reply ratio and sentiment analysis—as practical proxies for assessing shifts in public perception. This method provides a replicable, data-driven approach that can be applied to other IOs or public institutions facing similar crises in legitimacy. (4) The paper extends existing theories of IO legitimacy by focusing on popular legitimacy and using a computational social science approach. It suggests that digital engagement metrics can meaningfully reflect legitimacy in ways previously unexplored, offering a new lens through which to analyze public trust in global governance organizations.

In addition, the inclusion of sentiment analysis in our study (Multimedia Appendix 1) strengthens the interpretability of engagement trends. While engagement-based metrics capture public responsiveness, sentiment analysis allows us to understand changes in tone and framing in the WHO’s communications. Notably, the WHO adopted a more positive tone during the pandemic, but this did not correspond to a reversal in declining engagement or legitimacy signals. This suggests that tone alone is insufficient to repair or bolster institutional legitimacy during global crises. Instead, the interplay between message tone, content, and perceived trustworthiness may be more complex, requiring further investigation.

### Implications and Future Research

Regarding the implications of this study for readers, we highlight that (1) readers gain insight into how public sentiment toward the WHO has evolved, especially in response to crises such as COVID-19. Hence, they can better appreciate how digital engagement reflects wider public trust in or skepticism toward the organization’s authority. (2) The study underscores the importance of social media as a mirror of public sentiment, offering readers a new way to interpret digital interactions as reflections of public support or dissent. (3) By examining the relationship between an IO’s actions and public responses, readers can understand the complexities IOs face in maintaining legitimacy, particularly under the spotlight of global issues such as pandemics.

This study has practical implications for IOs and public institutions, demonstrating the value of monitoring social media engagement metrics to gauge public sentiment and adapt communication strategies in real time, particularly during crises. By developing systems that track digital engagement, IOs can enhance their responsiveness and transparency, fostering public trust. For research, the study introduces a new methodology for assessing popular legitimacy, paving the way for comparative analyses across IOs and crises and strengthening the intersection between computational social science and legitimacy studies. Future research can refine these metrics and expand the application of social media data to deepen our understanding of public trust in global governance.

### Limitations

This study is limited by our dataset of tweets. However, there are numerous OSNs with different engagement characteristics. Future studies can expand the research scope to compare different OSNs and study how they affect the popular legitimacy of the WHO and other IOs. In this regard, our methodology may also be used to analyze the popular legitimacy of other IOs and focus on crises or points in time that affect a given IO’s legitimacy. Another limitation of our study is the impact of changing X user demographics over time. For example, the number of teenagers on X has decreased in recent years due to the increased use of smartphones and the migration of this age group to TikTok, Instagram, and Snapchat [43]. As of April 2021, most X users were 25-34 years old [44]. It is unclear how these changes may impact our study’s conclusions. In addition, we clarify that while X replies often reflect critical engagement, they can also include positive feedback, and future studies should explore this variability in depth. In addition, the geographic origin of the tweets in the WHO dataset could not be determined, as the dataset does not include geographic metadata. Future research can apply our methodological framework to assess the popular legitimacy of IOs beyond the WHO, which was the focus of this study.

Moreover, our sentiment analysis is limited to the content of WHO tweets and does not include an analysis of user replies on X (Multimedia Appendix 1). Conducting sentiment analysis of user replies could capture the substance of public responses and provide valuable insights into the tone, framing, and reception of WHO communications. We encourage future research to explore this dimension.

In addition, future studies could expand our analysis using time series or multivariate models to assess how exogenous factors such as case counts, policy shifts, or media cycles influence patterns of digital engagement with IOs.

Beyond its specific insights into the WHO’s perceived legitimacy, our study speaks to a more fundamental shift in how IOs are scrutinized in the digital age. Public legitimacy is no longer mediated solely through traditional media or elite narratives, it unfolds in real time through dynamic, decentralized interactions on social platforms. As citizens increasingly evaluate global institutions based on web-based visibility and responsiveness, IOs must develop not only robust communication strategies but also analytics capacities to detect and respond to legitimacy shocks. The methodological framework introduced in our work suggests a path forward for evidence-based legitimacy management, enabling IOs to navigate complex political environments with greater agility and accountability. More broadly, our research highlights the need for global governance institutions to adapt in tandem with the evolving norms and expectations of digitally networked publics.

### Conclusions

The rise of social media platforms such as Facebook and X has given millions of users the ability to connect daily. These microcommunications are known to have the potential to impact people’s thinking and emotions, making them an attractive source of information for researchers investigating human conversation dynamics and the topics promoted by IOs [45].

In our case study using X, we aimed to develop an empirical measure of popular legitimacy based on user feedback. Our approach is unique on several fronts: we quantitatively analyze an OSN account of one of the largest IOs by exploiting the entire population, or over 13 years’ worth, of tweets from the account. By measuring the evolution of specific metrics and ratios, we demonstrate that the popular legitimacy of the WHO remained relatively stable until the onset of the COVID-19 pandemic. Once the pandemic hit, however, the IO saw its popular legitimacy diminish with no evidence of recovery through 2021. We hope the quantitative methodology we use in this study will be 1) used on other IOs over a similarly long period and 2) further developed to account for other factors that could play a role in the popular legitimacy of IOs, such as changes in the quantities of tweets.

To answer RQ1 we conducted a longitudinal analysis of the WHO’s tweets and audience engagement on X from 2008 to 2021, using metrics such as likes, retweets, and replies to track changes in public support. The study found that the WHO’s popular legitimacy was mostly stable before the COVID-19 pandemic, with some occasional fluctuations in engagement levels, before it notably decreased during the pandemic. This temporal analysis revealed clear trends in how the public’s perception of and engagement with the WHO shifted over time. In the case of RQ2, we highlight that by examining the engagement metrics specifically from the COVID-19 period (2020-2021), the study identified a significant drop in popular legitimacy. During this time, there was an increase in replies relative to retweets, suggesting heightened public scrutiny and possibly greater criticism. The research highlighted that while the WHO’s engagement spiked due to the pandemic, much of this attention was less supportive, indicating a decline in perceived legitimacy as the public became more vocal and critical of the WHO’s pandemic response. For RQ3, we used the retweet-to-reply ratio as a primary indicator of public sentiment, positing that an increase in replies was a greater indication of controversy or criticism than an increase in retweets. Additionally, sentiment analysis was applied to the WHO’s tweets to detect shifts in positive or negative tones over time. The study concluded that engagement metrics, especially the retweet-to-reply ratio, reliably indicated shifts in popular legitimacy and could serve as a valid proxy for public perception of an IO. Finally, to answer RQ4, we contextualized the WHO’s public engagement within broader theories of IO legitimacy, comparing our findings with existing research on public sentiment toward IOs during global crises. This contextualization demonstrated that the WHO’s challenges in maintaining legitimacy during the COVID-19 pandemic aligned with broader issues faced by IOs when under political scrutiny, supporting the idea that social media metrics can reflect public perception trends across global governance institutions.

## Appendix

Multimedia Appendix 1 Robustness check using sentiment analysis.

## Abbreviations

IO: international organization
OSN: online social network
RQ: research question
WHO: World Health Organization

## Data Availability

The dataset used in this study is publicly accessible on GitHub [46], in accordance with X’s data availability policy.
