# Supplementary material for: Social Media Metrics and Popular Legitimacy: Content Analysis of Pre– and Post–COVID-19 Public Engagement With the World Health Organization on X
Source: J Med Internet Res. 2025 Dec 8;27:e69959. doi: 10.2196/69959 (PMC12723361; doi:10.2196/69959)
Supplement: Multimedia Appendix 1 [file jmir_v27i1e69959_app1.docx]

# Appendix

**Robustness check using sentiment analysis**

As previously stated, there are two main approaches to measuring engagement based on OSNs and text-as-data: one based on ternary ratios and the other on the sentiment of the tweets.

Using specialized dictionaries containing terms that are positive, negative, or neutral, sentiment analysis determines the frequency of these terms over a wide variety of linguistic parameters in a given text or hashtags. To conduct sentiment analysis on the collected data, we employed the tidytext package in the R programming language, which is well-suited to the emotive nature of social media platforms, particularly X. Analyzing a text containing both positive and negative sentiment requires a reader to discern the presence and strength of each emotion.

Various methodologies and dictionaries exist to assess the sentiment or emotions conveyed in a text. The tidytext package provides access to multiple sentiment lexicons, including AFINN, Bing, and NRC. Each of these lexicons is based on unigrams, that is, single words that convey emotions. The lexicons contain a vast number of English terms, assigning them values based on the emotions they express, such as positive or negative sentiment, joy, anger, or sorrow.

The NRC lexicon categorizes words using a binary classification system (“yes/no”) into categories such as positive, negative, anger, anticipation, disgust, fear, joy, sadness, surprise, and trust. The Bing lexicon (Bing, 2012, 2015) also employs a binary classification system to divide words into positive and negative categories, with the former being more prevalent. We chose to utilize the AFINN lexicon for the sentiment analysis in our study, as it provides an overall score based on the volume of tweets received daily. The AFINN lexicon assigns a score to each word, ranging from -5 (strongly negative) to 5 (strongly positive), allowing for a more nuanced understanding of sentiment than that given by binary classification systems. The chosen methodology is effective for capturing general trends and patterns in sentiment over time, especially when focusing on large volumes of data. The lexicons are well-suited for high-level sentiment analysis of social media texts, where brevity often limits sentence complexity.

To calculate the daily sentiment score, we employed two methods: the total sum and the mean of the scores. In the first method, we aggregated the AFINN scores of all words within the tweets for each day. This approach provided us with a daily total sentiment score, which represented the overall emotional tone of the tweets on that particular day. A positive score indicated a predominantly positive sentiment, whereas a negative score suggested a predominantly negative one.

In the second method, we calculated the mean of the AFINN scores for each day, which involved summing the scores and then dividing by the total number of words in the tweets for that day. This approach yielded an average sentiment score, enabling us to gauge the general sentiment per word. By comparing the mean scores across different days, we were able to assess the variations in sentiment over time and identify potential trends or patterns in the public’s response to the WHO’s communications on X.

Figure S1 depicts the positive second derivative of retweets to visualize the accelerations of conversations per day. We also created a daily corpus aggregating all the texts from all the tweets posted in a day and ran our sentiment analysis on each daily corpus, which enabled us to easily visualize correlation levels between an acceleration of conversations and whether this acceleration was related to negative or positive sentiments.

Figure S1. *The p*ositive second derivative of retweets and the sum of sentiment associated with the tweet per day.


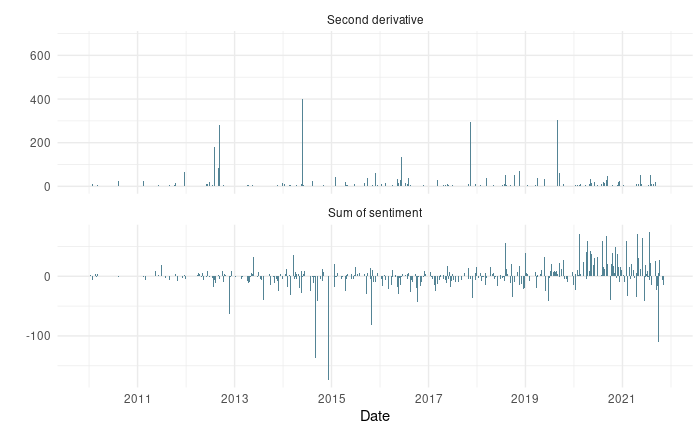


Figure S1 reveals, intriguingly, that despite a clearly concerted effort by the WHO to give a more positive tone to its tweets during the COVID-19 period, there was minimal acceleration of the conversation around these tweets. This finding suggests that increased positivity on the part of the IO has little impact (either positive or negative) on its popular legitimacy. Understanding these dynamics can help inform the organization’s communication strategies and contribute to the assessment of its legitimacy based on audience engagement on social media platforms.

Figure S2 presents the negative second derivative of retweets alongside the sum of sentiment associated with the daily corpus of text, which is created based on all the tweets of the day. This representation enables us to examine the relationship between the deceleration of retweets and the overall sentiment of the corresponding conversations.

By comparing these two metrics, we can gain insights into how the dynamics of audience engagement and the sentiment expressed in the conversations change over time. This analysis can help us understand whether negative sentiment is a driving factor in the deceleration of retweets or other factors are at play. Such insights contribute to our assessment of the organization’s legitimacy based on public engagement and perception on social media platforms.

**Figure S2.** Negative second derivative of retweets and the sum of sentiment associated with the tweet per day.


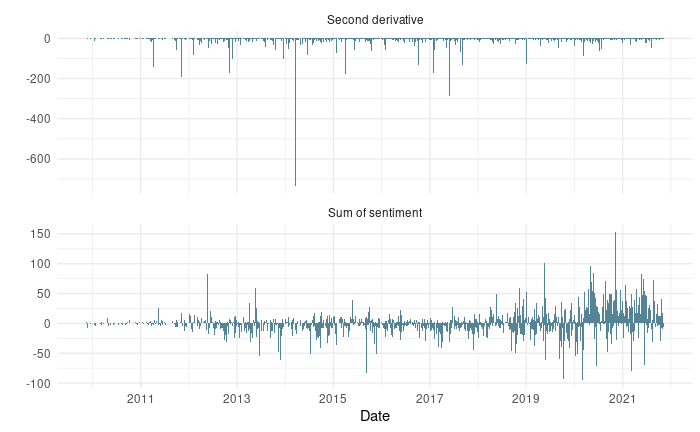


As in the positive sentiment analysis, there seems to be little correlation between the sum of sentiments and the negative second derivatives (i.e., the deceleration of a conversation). One possible explanation is that the overall quantity of tweeting affects the level of public engagement. Not only did the WHO tweet more positively in the final two years in the sample, but the number of tweets was over 10 times that of the first three years (i.e., 2008–2010). If tweeting is more frequent, then each tweet may carry less weight in the eyes of X users, thus inciting less variance in the reactions (meaning neither extreme accelerations nor decelerations).

In the subsequent figures, Figures S3 and S4, we shift our focus to analyzing the mean sentiment per day instead of the sum of sentiments. By examining the mean sentiment, we can account for variations in daily conversation volume and better understand the overall sentiment trend on a per-tweet basis. This approach allows us to capture more accurately the general mood of the audience and further investigate the relationship between sentiment and the pace of conversations. Ultimately, such an analysis can contribute to a more comprehensive understanding of the factors affecting the organization’s legitimacy and public perception on social media platforms.

**Figure S3.** The positive second derivative of retweets and the mean of sentiment associated with the tweet per day.


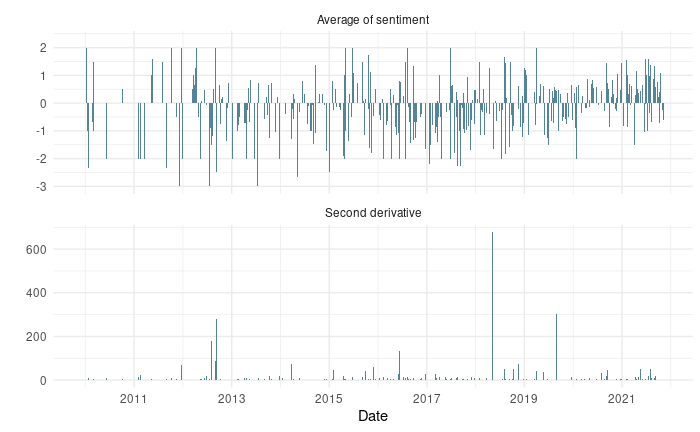


The pattern is similar to that outlined in Figures S1 and S2. There is a notably higher mean sentiment in the final two years of the sample (especially after the first half of 2020), but the impact this has on the positive second derivatives appears to be small in magnitude and does not differ greatly from that seen in previous years (despite those years having more negative sentiments on average).

Understanding this dynamic can provide valuable insights for organizations looking to enhance their legitimacy and public perception. By focusing on communicating messages that evoke positive sentiments, organizations may be able to encourage more active engagement, promote constructive conversations, and contribute to a favorable public image.

**Figure S4.** Negative second derivative of retweets and the mean of sentiment associated with the tweet per day.


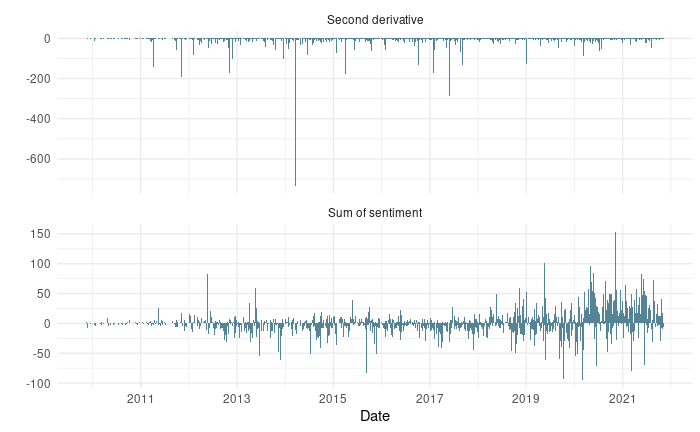


In conclusion, our analysis reveals that changes in the sum of sentiments of conversations seem to have little impact on the acceleration or deceleration of conversations. While it is beyond the scope of this paper to explore the possibility, it may be that the overall quantity of tweeting has a greater influence on the acceleration or deceleration of engagements than the sentiment of the original tweets. The strong increase in tweeting would also coincide with the changes we note in the retweet-to-reply ratio starting in 2020 and persisting through 2021.

These findings offer valuable insights for the research avenue proposed in this study, which focuses on using quantitative indicators to measure popular legitimacy based on text-as-data from OSNs. By further examining these dynamics, researchers may gain a more comprehensive understanding of how public sentiment and engagement patterns in OSNs can be utilized to assess the legitimacy of organizations such as the WHO, ultimately contributing to the development of more robust, data-driven methodologies in the realm of political science.
